# MATN1‐AS1 Promotes Tumour Metastasis and Sunitinib Resistance via E2F2 in Clear Cell Renal Cell Carcinoma

**DOI:** 10.1111/jcmm.70428

**Published:** 2025-02-25

**Authors:** Haibing Xiao, Mintian Fei, Qili Xu, Yu Gao, Rui Feng, Chaozhao Liang, Baojun Wang, Haolin Li

**Affiliations:** ^1^ Department of Urology The First Affiliated Hospital of Anhui Medical University Hefei China; ^2^ Anhui Provincal Key Laboratory of Urological and Andrological Diseases Research and Medical Transformation Anhui Medical University Anhui China; ^3^ Department of Urology Shuguang Hospital Affiliated to Shanghai University of Traditional Chinese Medicine Shanghai China; ^4^ Department of Urology The First Affiliated Hospital of Kunming Medical University Kunming China

**Keywords:** ccRCC, E2F2, EMT, lncRNA, MATN1‐AS1, sunitinib resistance

## Abstract

It has become increasingly recognised that MATN1‐AS1 is involved in multiple tumour development. The role of MATN1‐AS1 in clear cell renal cell carcinoma (ccRCC), however, is still largely unrecognised. This study investigated the molecular functions of MATN1‐AS1 in promoting ccRCC metastasis and sunitinib resistance. MATN1‐AS1 was found to be mainly located in the cytoplasm and was upregulated in ccRCC, and a positive association was seen between greater levels of MATN1‐AS1 expression and worse clinical outcomes. Downregulating MATN1‐AS1 significantly hindered cell proliferation, migration, invasion and epithelial‐mesenchymal transition (EMT). MATN1‐AS1 promoted tumour growth and metastasis in vivo. Mechanismly, MATN1‐AS1 targeted microRNA miR‐214‐5p, thereby upregulating E2F2 and promoting E2F2‐mediated EMT. We discovered that MATN1‐AS1 also promoted sunitinib resistance via E2F2 in vitro. Collectively, our research uncovered the protumor characteristics of MATN1‐AS1 and suggested it as a therapeutic target for reverse sunitinib resistance in ccRCC.

## 1 Introduction

In the genitourinary malignancies field, Renal cell carcinoma (RCC) is a frequently observed one [1]. Among all kinds of RCC, clear cell renal cell carcinoma (ccRCC) is the most prevalent subtype [2]. The early diagnosis of RCC is challenging, and the high recurrence rate is the main obstacle to RCC treatment [3]. While targeted drugs such as sunitinib demonstrate temporary inhibition of tumour progression, the enduring issue of resistance contributes to a persistently high mortality rate in RCC [4, 5]. To enhance the prognosis of ccRCC, novel biomarkers and therapeutic targets should be identified.

As a specific classification of RNAs, long noncoding RNAs (lncRNAs) have no or only weak protein encoding potentials [6]. However, multiple investigations have illustrated the pivotal function of lncRNAs in tumour development, including key aspects like tumour metastasis and drug resistance [7, 8, 9]. Numerous studies have strengthened the possibility that lncRNAs could be a potential therapeutic vulnerability in malignancy treatment [10]. A newfound lncRNA, MATN1 antisense RNA 1 (MATN1‐AS1), was first reported in ischemic stroke [11]. Recently, MATN1‐AS1 has been garnering increasing attention across multiple malignancies. By functioning as a sponge of miRNAs, MATN1‐AS1 could promote tumour progression [12, 13, 14]. However, the potential effects of MATN1‐AS1 in ccRCC still need investigation.

Epithelial‐mesenchymal transition (EMT) is a crucial cellular program for malignant progression. In this process, epithelial cells gradually lose their epithelial appearance and transition into a mesenchymal phenotype, resulting in downregulating of E‐cadherin and upregulating of N‐cadherin, Vimentin [15]. Also, some EMT‐promoting transcription factors, such as SNAIL, SLUG, and TWIST1, could be overexpressed [15, 16].

In our study, MATN1‐AS1 was upregulated in ccRCC and was correlated with worse clinical outcomes. In addition, downregulating MATN1‐AS1 inhibited cell malignancy phenotypes. Upon conducting more research, it was discovered that by upregulating E2F2, MATN1‐AS1 may promote EMT and sunitinib resistance in ccRCC. Remarkably, targeting MATN1‐AS1 showed promising effects in hindering tumour metastasis. In conclusion, our research suggests that MATN1‐AS1 can serve as a prognostic factor and drug target for ccRCC patients.

## 2 Materials and Methods

### 2.1 Data Preparation

The gene expression data and phenotype data for a cohort of 613 patients diagnosed with ccRCC (TCGA‐KIRC cohort) were acquired from The Cancer Genome Atlas (TCGA) database (https://portal.gdc.cancer.gov/, September 1, 2023). R package “TCGAbiolinks” was used to download and prepare RNAseq and clinical data [17]. As for datasets from the GEO database, pre‐normalised data were directly downloaded using the R package “GEOquery” [18].

### 2.2 Investigation of Gene Expression Differences and Prognosis Analysis

MATN1‐AS1 expression discrepancies were calculated with Wilcoxon analysis and depicted with the R package “ggpubr” in R‐4.2.1. Prognostic values of MATN1‐AS1 and other clinical features were evaluated using Kaplan–Meier algorithms, Receiver operating characteristic curves (ROC), and Cox regression analysis. First, individuals were categorised into two clusters based on MATN1‐AS1's median expression value. Then, a prognosis model was generated with the “survival” R package and plotted with the “survminer” modules in R‐4.2.1. The R package “timeROC” was utilised to evaluate the prognostic value of the built model. The nomogram survival spectrum was constructed and verified with R‐4.2.1 using multivariate Cox regression analysis method.

### 2.3 Patient Samples Collection

Fifty‐two pairs of human ccRCC tissues and adjacent normal tissues were obtained during 2021‐2024. The basic clinical characteristics were: Age, > 57 (*n* = 27), <=57 (*n* = 25); Gender, Male (*n* = 36), Female (*n* = 16); Clinical Stage, I (*n* = 39), II (*n* = 11), III (*n* = 2), IV (*n* = 0); T Stage, T1 (*n* = 40), T2 (*n* = 11), T3 (*n* = 1). Samples were obtained from the First Affiliated Hospital of Anhui Medical University (Hefei, China). These specimens were identified as ccRCC by the pathological department based on pathology reports. Collected tissues were transferred with liquid nitrogen. This study followed the ethical guidelines of the Declaration of Helsinki. It was approved by the Ethics Committee of Human Research of The First Affiliated Hospital of Anhui Medical University (PJ2019‐14‐22), and all patients signed informed consent forms.

### 2.4 Real‐Time RT‐PCR (RT‐qPCR)

The samples were obtained using TRIzol (no. 15596026, ThermoFisher, USA). Concentrations of the obtained samples were measured with NanoDrop 2000 (no. ND‐2000, NanoDrop Technologies, USA). Subsequently, the RNA underwent reverse transcription using the PrimeScript RT kit (no. RR037A, Takara, Japan). After that, we conducted qPCR with SYBR Green Mix (no. RR820A, Takara, Japan) and obtained results on the ABI7500 platform (no. 4351105, ThermoFisher, USA). For data analysis, GAPDH was used as a normalisation reference. Primers were listed as follows: MATN1‐AS1: 5′‐CTTCACGGTGCTGGCATAGTT‐3′, 5′‐GGACAGCATTGCGTTTCTCAC‐3′; E2F2: 5′‐CGTCCCTGAGTTCCCAACC‐3′, 5′‐GCGAAGTGTCATACCGAGTCTT 3′; GAPDH: 5′‐TTGCCCTCAACGACCACTTT‐3′, 5′‐TGGTCCAGGGGTCTTACTCC‐3′. The whole procedure was executed in accordance with the guidelines provided by the manufacturer.

### 2.5 RNA Fish

The MATN1‐AS1 RNA fluorescence in situ hybridization (FISH) technique was conducted using FISH probes that were specifically developed and produced by Ribobio (Ribobio Technologies, Guangzhou, China). Cells were seeded onto glass coverslips measuring Φ 15 mm, then fixation using a 4% (w/v) paraformaldehyde solution for 10 min. Subsequently, the cells were permeabilized using a solution of 1% Triton X‐10 diluted in PBS at 4°C. After 10 min incubations, coverslips were treated using 200 μL pre‐hybridization buffer (no. C10910, Ribobio Technologies, Guangzhou, China) under 37°C for a duration of 30 min. Then, they were treated using the hybridization buffer (no. C10910, Ribobio Technologies, Guangzhou, China) containing 250 nM FISH probes targeting U6, 18S, or MATN1‐AS1 protected from light at 37°C overnight. Subsequently, samples underwent a series of washing steps. Specifically, they were subjected to three consecutive washes with Wash Buffer I, each lasting for a duration of 5 min. Then, a single wash with Wash Buffer II was performed, followed by a final wash with Wash Buffer III. All processes were performed in the dark at 42°C. After mounting slices onto microscope slides using ProLong Glass mounting media (no. P36980, ThermoFisher, USA), photographs were obtained using a Zeiss confocal microscope.

### 2.6 Cell Culture

Cells were acquired from Procell (Wuhan, China) in September 2019 and were authenticated by STR analysis. The cells were maintained using RPMI‐1640 medium (no. PM150110, Procell, Wuhan, China) with 10% FBS (no. SH30071.03, HyClone, USA) and 1% penicillin–streptomycin (no. SV30010, Cytiva, USA) added. Meanwhile, ACHN and A498 cells were maintained using MEM (no. PM150410, Procell, Wuhan, China). Cells were grown with 5% CO2 supplied at 37°C. A498 cells were subjected to a gradual increase in sunitinib (no. HY‐10255A, MCE, Shanghai, China) dosage, reaching a maximum concentration of 10 μM over 1 month to establish a sunitinib‐resistant cell line. No mycoplasma contamination was found in all cell lines.

### 2.7 MATN1‐AS1 Knocking Down

Lentivirus for MATN1‐AS1 knocking down was constructed by GeneChem Co. Ltd. (Shanghai, China). Transfections were conducted using the Transfection Kit (no. LPK001, GeneChem, China) following the manufacturer's protocol.

### 2.8 Cell Proliferation Assay

Before measurement, old culture mediums were removed, and fresh medium with 10% CCK‐8 (no. C0038, Beyotime, China) was added in the dark. After reacting in the incubator for 1 h, the OD450 absorbance values were measured with TECAN Infinite 200 PRO (Tecan, Switzerland). EdU was performed with a Click‐on EdU kit (no. C6045M, Uelandy, China). The cells were incubated with a concentration of 10 μM EdU for 10 h. Subsequently, they were treated with a 4% PFA solution for a duration of 10 min at ambient temperature. Cells were stained using the Click‐iT EdU kit for fluorescence staining.

### 2.9 Transwell Assay

The experiment used an 8 μm pores Transwell chamber (no. 3422, Corning, USA). As for invasion assays, the membranes should be additionally coated in Matrigel (no. 356234, BD Biosciences). Before subsequent experimentation, cells were resuspended using an FBS‐free medium. 700 μL of MEM containing 10% FBS was introduced into the bottom chambers. Subsequently, 200 μL of cell suspensions were introduced into the upper chambers. After being placed for 24 h, cells were treated with 4% paraformaldehyde solution. After wiping off cells remaining in the upper chamber, cells were stained using 0.01% crystal violet. Three random pictures were imaged with OLYMPUS IX71 (Olympus, Japan) and calculated using ImageJ‐1.53 t.

### 2.10 Mouse Tumour Xenograft Module

GemPharmatech Co. Ltd. (Nanjing, China) supplied the male BALB/C nude mice. Next, 1 × 10^7^ 786‐O cells were resuspended in 100 μL PBS with Matrigel (no. 0827045, ABW, Shanghai, China) 1:1 added. The animals were randomly separated into accordance groups, and the suspensions were then administered by subcutaneous injection on the left side of the mice. The xenograft volume was determined using the following method: V = (D × d^2^)/2. After 4 weeks, mice were sacrificed, and tumours were imaged. As for the tumour metastasis model, we injected 5 × 105 cells suspended in 50 μL PBS per mouse for the tumour metastasis model. Results were then acquired with Tanon ABL‐X6 Pro (Tanon Co. Ltd., Shanghai, China) after 6 weeks. This experiment was approved by the Institutional Animal Care and Use Committee of Anhui Medical University (LLSC20221265).

### 2.11 Western Blotting Assay

Samples were extracted using RIPA reagent, which added 1 mM protease and phosphatase inhibitor mixture (no. P1045, Beyotime, Shanghai, China). Next, we mixed them with SDS‐PAGE loading buffer (no. LC2676, Invitrogen, USA), then heated them for 5 min. PVDF membrane (no. IPFL20200, Millipore, Germany) and 12.5% SDS‐PAGE gel were used for sample separation and transference. Subsequently, the membranes were obstructed using a 5% bovine serum albumin (no. ST023, BSA, USA) solution. Following the appropriate antibodies' incubation, membranes were reacted with anti‐goat secondary antibodies (1:3000; SA00001‐4; Proteintech). After extensive washing, membranes were reacted with BeyoECL Moon Chemiluminescence Substrate (no. P0018FS, Beyotime, Shanghai, China). Antibody reagents were anti‐E2F2 (1:1000; bsm‐52641R; Bioss), anti‐SNAI1 (1:1000; 13099‐1‐AP; Proteintech), anti‐SNAI2 (1:1000; 12129‐1‐AP; Proteintech), anti‐Vimentin (1:1000; 10366‐1‐AP; Proteintech), anti‐N‐cadherin (1:1000; 22018‐1‐AP; Proteintech), anti‐E‐cadherin (1:1000; no. 3195; CST), and anti‐GAPDH (1:2000; no. 2118; CST). GAPDH was detected as a reference for normalisation. The results were acquired with a CLiNX ChemiScope 5000 imager.

### 2.12 Gene Set Enrichment Analysis (GSEA)

The GSEA program (https://www.gsea‐msigdb.org/gsea/msigdb, September 1, 2023) was used to execute comprehensive procedures. Individuals were first categorised into two classifications based on their MATN1‐AS1 median values. Following that, patients were ranked by MATN1‐AS1 mRNA expression. Then, we performed GSEA between these two groups in the KEGG gene set (https://www.gsea‐msigdb.org/gsea/msigdb/human/genesets.jsp?collection=CP:KEGG_LEGACY).

### 2.13 RNA Sequencing

The experiment was performed on cells with MATN1‐AS1 downregulation and relative controls. OE Biotech Co. Ltd. performed RNA sequencing. Then, the DESeq2 R package was used to identify differential expression genes, with DEGs set as |LogFC| > 1.

### 2.14 Luciferase Analysis

293 T cells were first cultured in 96 well plates, followed by 100 nM luciferase reporter vector and 100 nM miR‐214‐5p co‐transfected. After 48 h of transfection, cells were lysed, and supernatants were treated with a Dual‐Luciferase Reporter Kit (no. RG027, Beyotime, China). Results were acquired on the Infinite 200 PRO reader (TECAN, Switzerland).

### 2.15 Data Analysis

The statistical analysis was conducted using GraphPad Prism or R‐4.2.1 software. Detailed methods and the number of animals or samples are stated in the article.

## 3 Results

### 3.1 MATN1‐AS1 Is Up‐Regulated in ccRCC and Associated With Worse Clinical Outcomes

**FIGURE 1:**
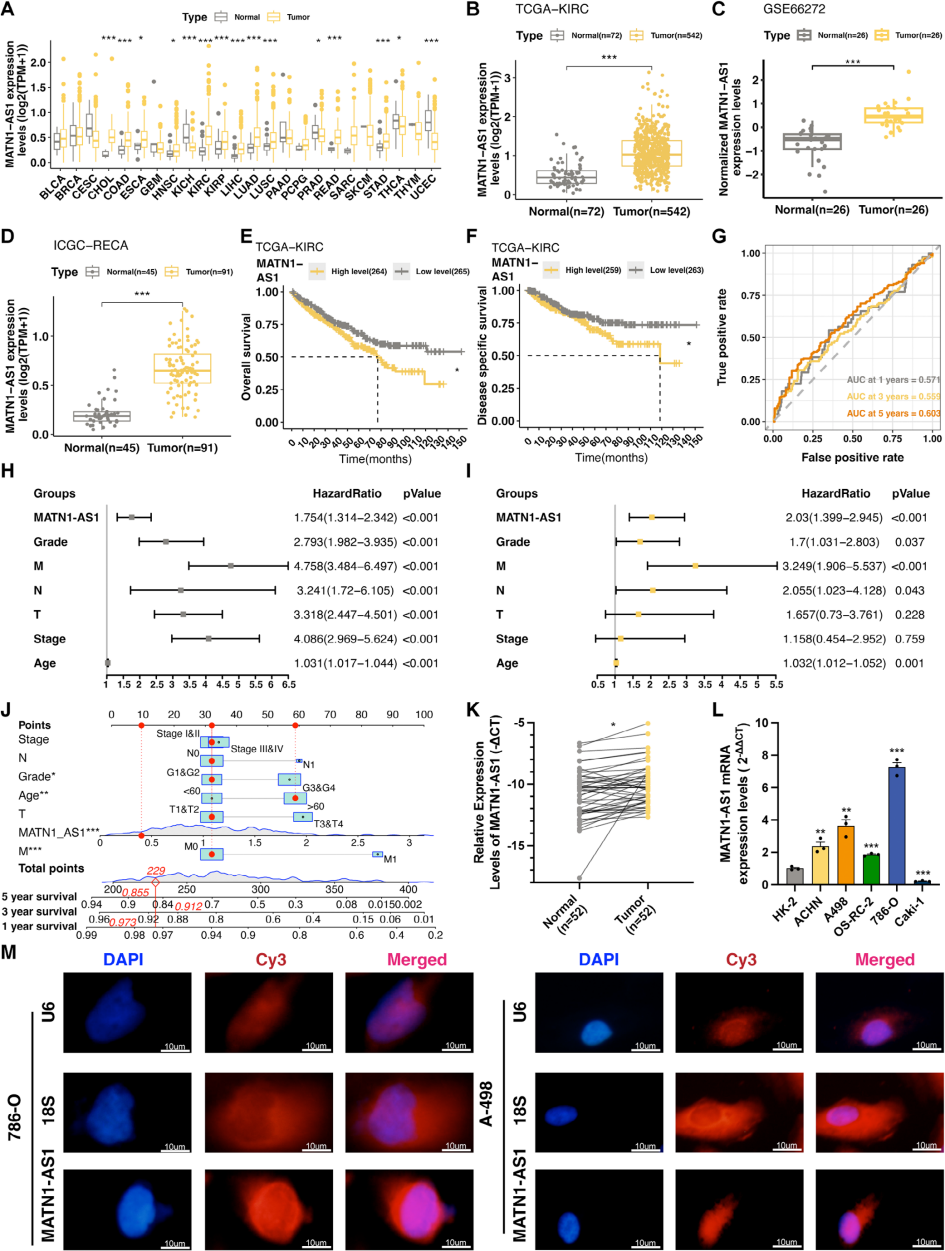
MATN1‐AS1 expression and clinical landscapes in ccRCC. (A) Expression levels of MATN1‐AS1 between malignant and normal tissues in diverse cancers (Data were normalised with log_2_(TPM + 1), Means ± SEM, Wilcoxon test, *p < 0.05, ***p < 0.001). (B) Expression level of MATN1‐AS1 between ccRCC and normal tissues in TCGA‐KIRC dataset (Data were normalised with log_2_(TPM + 1), Means ± SEM, Wilcoxon test, ***p < 0.001). (C) MATN1‐AS1 expression levels in paired ccRCC and normal tissues in the GSE66272 cohort (Data were normalised with log_2_(TPM + 1), Mean ± SEM, Wilcoxon test, ***p < 0.001). (D) MATN1‐AS1 expression levels in ccRCC and normal tissues in the ICGC‐RECA dataset (Data were normalised with log_2_(TPM + 1), Mean ± SEM, Wilcoxon test, ***p < 0.001). (E) Overall survival (OS) curves of MATN1‐AS1 in ccRCC (Log‐rank test, *p < 0.05). (F) Disease‐specific survival (DSS) curves of MATN1‐AS1 in ccRCC (Log‐rank test, *p < 0.05). (G) Time‐dependent ROC analysis of MATN1‐AS1 in ccRCC. (H) Univariable Cox regression analysis and (I) Multivariable Cox regression analysis of the correlation between patients' overall survival outcomes and MATN1‐AS1 expression levels and other clinical variables (Cox regression analysis). (J) Nomogram module constructed based on the multivariable Cox regression analysis. (K) MATN1‐AS1 RNA expression levels in paired ccRCC tissues and adjacent normal renal cortex detected by RT‐qPCR method (Student's *t*‐test, ***p < 0.001). (L) MATN1‐AS1 expression levels in renal cancer cell lines identified by RT‐qPCR (Mean ± SEM, Student's *t*‐test, **p < 0.01, ***p < 0.001). (M) FISH detection of the MATN1‐AS1 subcellular location in 786‐O and A‐498 cell lines.

We first evaluate MATN1‐AS1 expression levels from the pan‐cancer perspective. Our findings showcased conspicuous down‐regulation of MATN1‐AS1 in most cancers, particularly in gynaecological oncology, such as cervical cancer (CESC), endometrioid cancer (UCEC), ovarian cancer (OV), and uterine carcinosarcoma (UCS), indicating its possible tumour suppressor role in these malignancies (Figure 1A). However, a notable overexpression of MATN1‐AS1 was found in ccRCC tissues from three independent cohorts (Figure 1B–D). Also, high MATN1‐AS1 expression individuals showcased a worse clinical outcome, including overall survival (OS) rates and disease‐specific survival (DSS) rates (Figure 1E,F). We hypothesize that MATN1‐AS1 may serve as a pro‐cancer gene in ccRCC patients. Next, we conducted a Cox regression module to test whether MATN1‐AS1 could be a potential prognosis biomarker. According to the time‐dependent ROC curve, MATN1‐AS1 expression levels could be used to predict patients' five‐year survival to some extent (Figure 1G). MATN1‐AS1 also emerged as an independent prognostic factor, as depicted in the forest plot (Figure 1H,I). When analysing the relationship between MATN1‐AS1 expression level and clinical parameters, we noticed a significant link between MATN1‐AS1 and tumour metastasis state (Table 1). Finally, we constructed a nomogram module to predict patients' survival time (Figure 1J).

**TABLE 1:** Correlation between MATN1‐AS1 mRNA expression and clinicopathological parameters of ccRCC patients.

	Number	MATN1‐AS1 mRNA expression level		*p*
		High(*n* = 264)	Low(*n* = 265)	
Age (Years; Median, 25th and 75th percentile)	61.00 (52.00, 70.00)	60.000 (51.000, 69.000)	61.000 (53.000, 70.000)	0.3474
T stage (%)				
T1	269	128 (48.48)	141 (53.21)	0.3069
T2	69	38 (14.39)	31 (11.70)	
T3	180	90 (34.09)	90 (33.96)	
T4	11	8 (3.03)	3 (1.13)	
N stage (%)				
N0	239	119 (45.08)	120 (45.28)	0.3037
N1	16	5 (1.89)	11 (4.15)	
NX	274	140 (53.03)	134 (50.57)	
M stage (%)				
M0	420	195 (73.86)	225 (84.91)	< 0.0001*
M1	79	43 (16.29)	36 (13.58)	
MX	30	26 (9.85)	4 (1.51)	
G grade (%)				
G1	13	11 (4.17)	2 (0.75)	0.0636
G2	228	117 (44.32)	111 (41.89)	
G3	205	98 (37.12)	107 (40.38)	
G4	75	36 (13.64)	39 (14.72)	
GX	8	2 (0.76)	6 (2.26)	
TNM stage (%)				
Stage I	263	127 (48.11)	136 (51.32)	0.7401
Stage II	57	30 (11.36)	27 (10.19)	
Stage III	123	59 (22.35)	64 (24.15)	
Stage IV	83	46 (17.42)	37 (13.96)	
Unreported	3	2 (0.76)	1 (0.38)	

*Note:* ^*^means statistal significant.

To validate our findings based on bioinformatics analysis, we detected MATN1‐AS1 RNA expression levels in several paired ccRCC and normal specimens. Consistent with results from the external dataset, MATN1‐AS1 was up‐regulated in ccRCC samples (Figure 1K). Also, MATN1‐AS1 expression level was higher in renal cancer cell lines, except for Caki‐1 (Figure 1L). All these findings indicated the potential oncogene role of MATN1‐AS1 and suggested it to be a prognostic biomarker for ccRCC patients.

Investigating the subcellular location of lncRNAs is pivotal in elucidating their functions, as they predominantly regulate various cellular processes by interacting with other molecules [19]. To determine the subcellular location of MATN1‐AS1, we employed the lncRNA fluorescence in situ hybridization (FISH) assay. Our results showed that MATN1‐AS1 primarily resides in the cytoplasm of 786‐O and A‐498 cells (Figure 1M).

### 3.2 Downregulating MATN1‐AS1 Hindered Tumour Development in ccRCC

**FIGURE 2:**
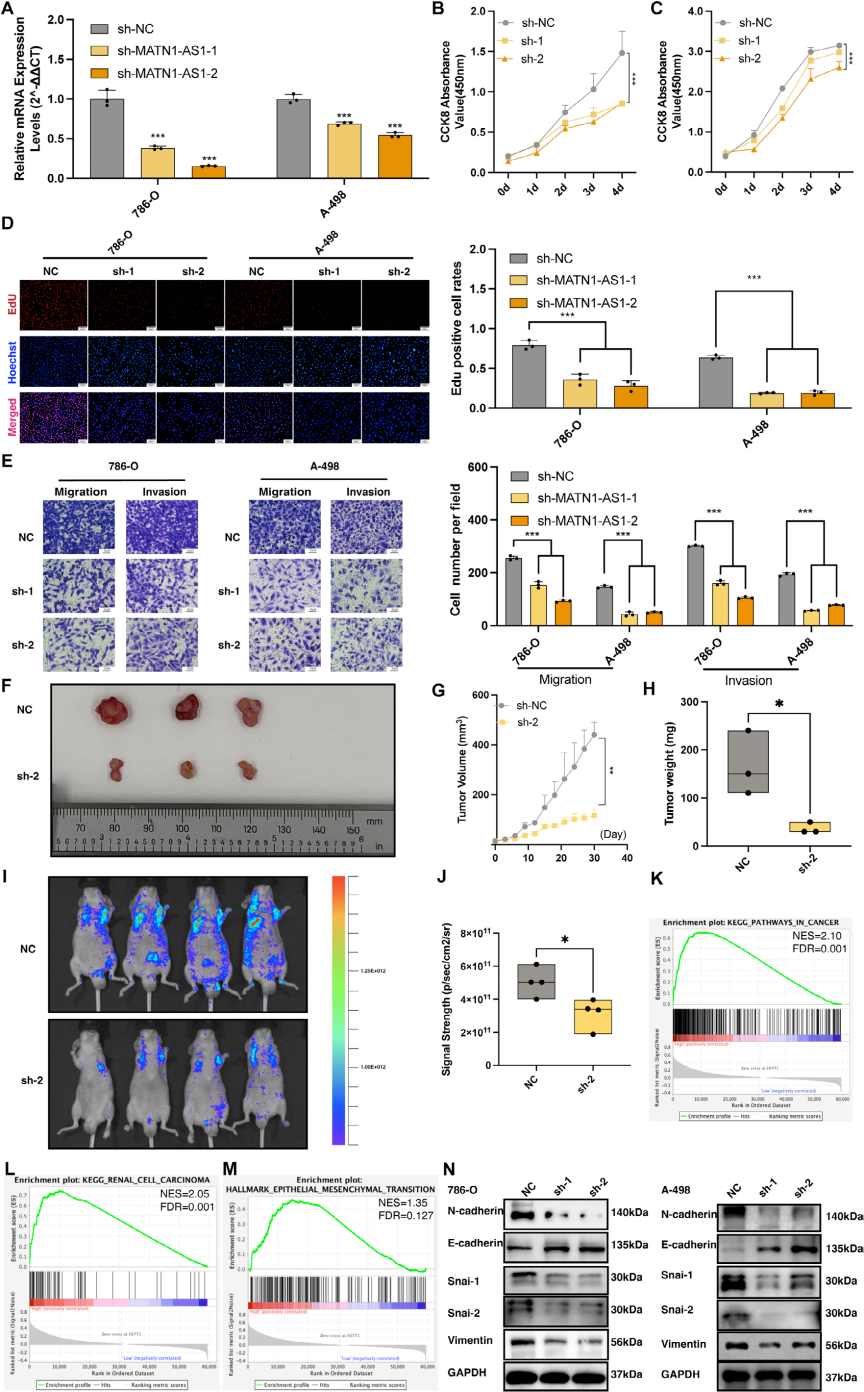
Knocking down MATN1‐AS1 regressed tumour formation, proliferation, and metastasis in ccRCC. (A) RT‐qPCR revealed the successful knocking down of MATN1‐AS1 in 786‐O and A‐498 cell lines (Mean ± SEM, Student's *t*‐test, ***p < 0.001). (B–D) Knocking down MATN1‐AS1 inhibited cell proliferation in 786‐O and A‐498 cell lines (Two‐way repeated‐measures analysis of ANOVA with Geisser–Greenhouse correction and one‐way ANOVA, ***p < 0.001). (E) Knocking down MATN1‐AS1 downregulated cell migration and invasion abilities in 786‐O and A‐498 cell lines (One‐way ANOVA, ***p < 0.001). (F–H) Tumour volumes, growth rates, and tumour weights were tremendously smaller in the MATN1‐AS1 knockdown group compared to the control group. (I) Knocking down MATN1‐AS1 relieved tumour metastasis in vivo, and results were measured as (J) signal strength (p/s/cm2/sr, Mean ± SEM, Student's *t*‐test, *p < 0.05). (K–M) Gene set enrichment analysis (GSEA) of high‐ and low‐MATN1‐AS1 expression groups. (N) Expression level changes of EMT markers after knocking down MATN1‐AS1 in 786‐O and A‐498 cell lines.

Given that MATN1‐AS1 was significantly upregulated in 786‐O and A‐498 cells, we authorised these two cell lines for further study. We knocked down MATN1‐AS1 in cancer cells with two sequences (Figure 2A). Our results suggested that downregulating MATN1‐AS1 could inhibit cell proliferation capability (Figure 2B–D). Moreover, cell migration and invasion abilities were significantly hindered (Figure 2E). The cell‐derived xenograft module showed that knockdown MATN1‐AS1 hindered tumour formation, as tumour volume (Figure 2F), tumour growth ability (Figure 2G), and tumour weight (Figure 2H) were all decreased compared to the control group. Next, we injected either control groups or MATN1‐AS1 downregulated cells into the mouse tail vein, and the results showed limited tumour metastasis ability (Figure 2I,J).

In the development of malignancies, epithelial‐mesenchymal transition (EMT) is an essential hallmark for promoting tumour metastasis [15, 20]. During this, epithelial cells gradually shed their cell–cell junctions and obtain malignancy features [15]. Considering the significant inhibition of tumour metastasis induced by MATN1‐AS1 knockdown, we checked the potential link between MATN1‐AS1 and EMT. In gene set enrichment analysis (GSEA), cancer‐related pathways, renal cell carcinoma pathways, and EMT were upregulated in high MATN1‐AS1 expression groups (Figure 2K–M). Also, EMT markers showed significant changes following MATN1‐AS1 knockdown. As depicted in Figure 2. N, mesenchymal marker N‐cadherin, and other EMT‐related proteins were inhibited, while epithelial marker E‐cadherin was upregulated.

### 3.3 MATN1‐AS1 Regulates E2F2 Expression in ccRCC

**FIGURE 3:**
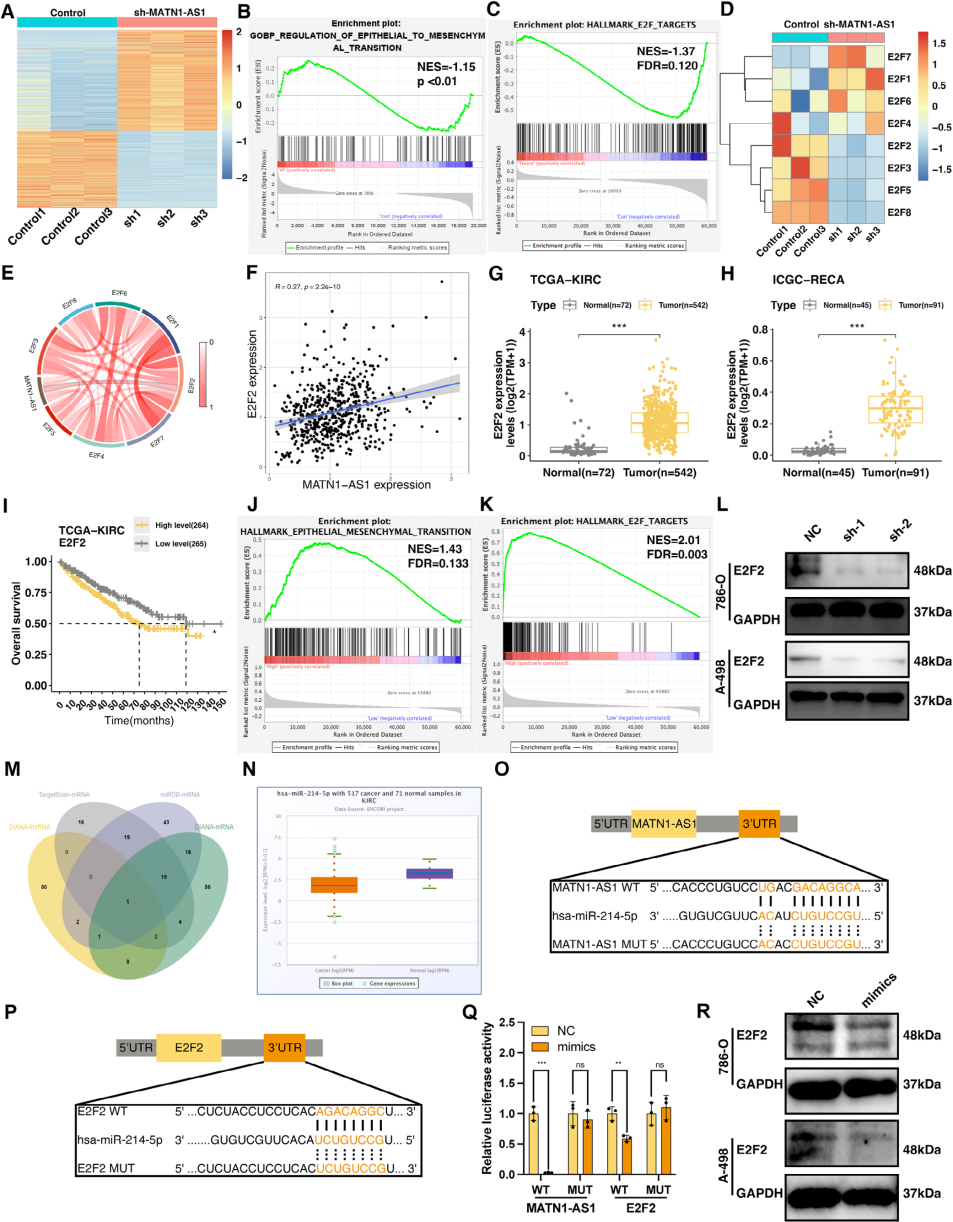
MATN1‐AS1 regulates E2F2 expression in ccRCC. (A) Heatmap showing DEGs expression profiles of 786‐O cells transfected with control (Control 1–3) or MATN1‐AS1 targeted sequence (sh 1–3). Data were normalised with FPKM. Results were filtered with *p* < 0.05, |log_2_FC| > 1. (B, C) GSEA of Control and MATN1‐AS1 knocked down groups. (D) Heatmap showing the expression level changes of the E2F family after knocking down MATN1‐AS1. Data were normalised with FPKM. (E) Co‐expression analysis of E2Fs with MATN1‐AS1 in TCGA‐KIRC dataset (Pearson correlation test). (F) Expression correlation between E2F2 and MATN1‐AS1 in TCGA‐KIRC dataset (Pearson correlation test). (G) E2F2 expression levels in ccRCC and normal tissues in the TCGA‐KIRC dataset (Data were normalised with log_2_(TPM + 1), Mean ± SEM, Wilcoxon test, ***p < 0.001). (H) E2F2 expression levels in ccRCC and normal tissues in the ICGC‐RECA dataset (Data were normalised with log_2_(TPM + 1), Mean ± SEM, Wilcoxon test, ***p < 0.001). (I) Overall survival (OS) curves of E2F2 in ccRCC (Log‐rank test, *p < 0.05). (J, K) GSEA between High‐ and Low‐E2F2 expression individuals from TCGA‐KIRC dataset. (L) E2F2 expression level changes after knocking down MATN1‐AS1. (M) Potential micro‐RNA links between MATN1‐AS1 and E2F2. (N) miR‐214‐5p expression levels in ccRCC and renal tissue from TCGA‐KIRC dataset (*p* = 2.2 × 10^−13^). (O, P) Binding sites schematic diagrams of miR‐214‐5p with E2F2 and MATN1‐AS1. (Q) Dual‐luciferase reporter assay results of MATN1‐AS1 and E2F2 (Mean ± SEM, Student's *t*‐test, ns, no significance, **p < 0.01, ***p < 0.001). (R) E2F2 expression level changes after being treated with miR‐214‐5p mimics.

Next, we performed next‐generation sequencing (NGS) to elucidate the gene expressions and signalling pathways altered by MATN1‐AS1 knockdown, and 2997 genes were identified (Figure 3A, Table S1). As illuminated by GSEA enrichment analysis, EMT regulatory pathways and E2F target pathways were both inhibited in MATN1‐AS1 downregulated cells (Figure 3B,C). Indeed, downregulating MATN1‐AS1 resulted in several E2Fs inhibitions (Figure 3. D). Among all these E2Fs, E2F2 showed the strongest correlation with MATN1‐AS1 in the TCGA‐KIRC cohort (Figure 3E,F, Table S2). Also, E2F2 was highly expressed in ccRCC tissues (Figure 3G,H) and associated worse OS rates (Figure 3I). Our GSEA analysis results suggest the EMT and E2F signalling pathway enrichment in high E2F2 expression samples (Figure 3J,K). Moreover, E2F2 has been proven to be an EMT inducer and metastasis promoter in multiple malignancies, including ccRCC [21, 22, 23]. In accordance with the mRNA expression level changes, downregulating MATN1‐AS1 also hindered E2F2 expression (Figure 3L). These results indicated that MATN1‐AS1 may promote EMT through regulating E2F2.

### 3.4 MATN1‐AS1 Promotes E2F2‐Mediated EMT by Sponging miR‐214‐5p

For cytoplasm located lncRNA, a commonly seen signalling regulate mechanism is competing endogenies RNA (ceRNA); that is, lncRNA “absorbed” a micro‐RNA that targeted downstream mRNA, thereby promoting gene expression [24]. MATN1‐AS1 may also promote E2F2 expression by functioning as a ceRNA. To validate our above hypothesis, we predicted MATN1‐AS1 and E2F2 targeted micro‐RNAs from four independent databases, and the intersection showed one micro‐RNA named miR‐214‐5p (Figure 3M). miR‐214‐5p was lower in malignancy tissues compared to the control groups (Figure 3N). Next, we mutated the targeted site in the 3′ UTR and performed luciferase analysis (Figure 3O,P). After conducting mutation to the seed sequences at the 3′ UTR, the suppressing abilities of miR‐214‐5p were significantly abolished (Figure 3Q).

**FIGURE 4:**
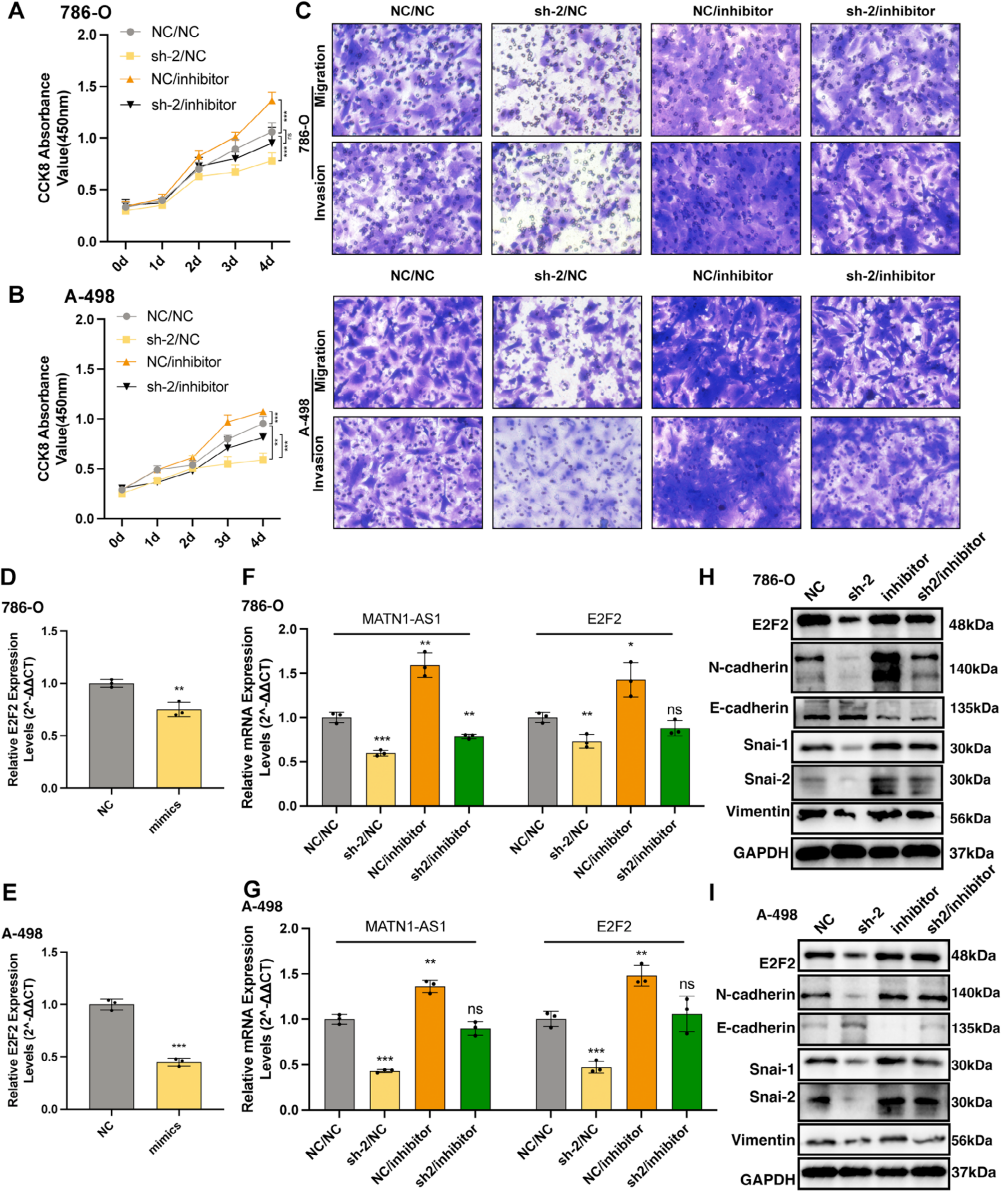
MATN1‐AS1/miR‐214‐5p/E2F2 axis regulated EMT in ccRCC. (A, B) Cell viability of indicated groups measured with CCK8 assay (Mean ± SEM, Two‐way repeated‐measures analysis of ANOVA with Geisser–Greenhouse correction, ns, no significance, **p < 0.01, ***p < 0.001). (C) Cell migration and invasion abilities of corresponding groups as measured with the Transwell assay. (D, E) RNA expression level changes of E2F2 after overexpression miR‐214‐5p in 786‐O and A‐498 cell lines. (F, G) RNA expression levels of E2F2 and MATN1‐AS1 in indicated cells. (H, I) E2F2 and EMT biomarkers expression changes across four groups.

After overexpressing miR‐214‐5p in 786‐O and A‐498 cell lines, expression levels of E2F2 were obviously inhibited, as detected by the RT‐qPCR and WB assays (Figure 3R, Figure 4D,E). When inhibited miR‐214‐5p, both MATN1‐AS1 and E2F2 expression levels were downregulated (Figure 4F–I). Similarly, EMT‐related markers showed the same trend. To be clear, the upregulating of N‐cadherin, Snai‐1, Snai‐2 and Vimentin and the downregulating of E‐cadherin (Figure 4F–I). In contrast, downregulated E2F2 expression after knocking MATN1‐AS1 could be partly revised by inhibiting miR‐214‐5p (Figure 4F–I).

**FIGURE 5:**
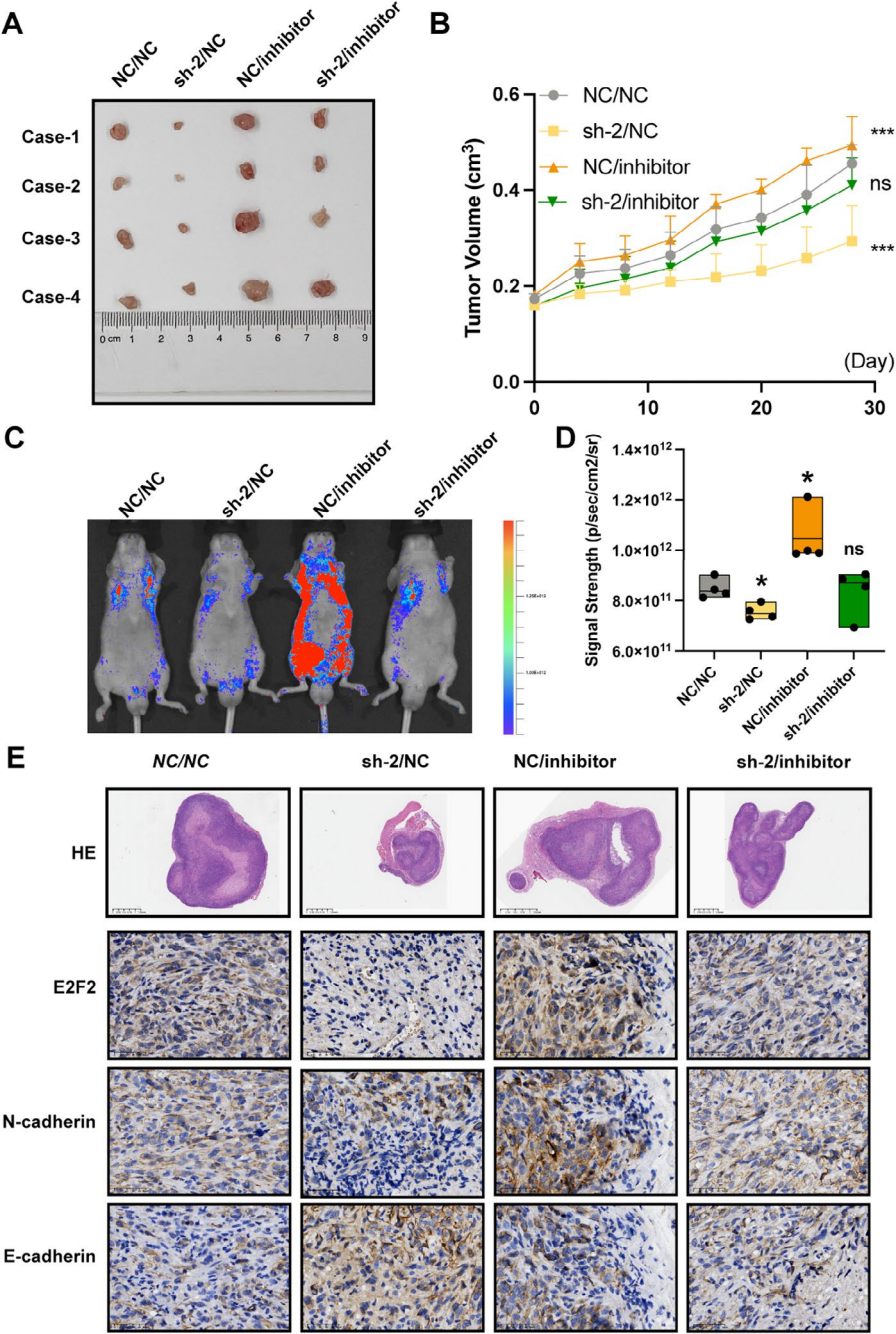
MATN1‐AS1/miR‐214‐5p/E2F2 axis affects tumour metastasis in ccRCC. (A) Four groups of nude mice were subcutaneously injected with response cells, and tumours were removed and imaged. (B) Tumour volumes were measured every 3 days and drawn as a curve plot (Mean ± SEM, Two‐way repeated‐measures ANOVA with Dunnett multiple comparisons test correction, ns, no significance, ***p < 0.001). (C, D) Four groups of nude mice were tail vein‐injected corresponsive cells and imaged after 6 months (Signals were normalised as p/s/cm2/sr, Mean ± SEM, Student's *t*‐test, ns, no significance, *p < 0.05). (E) E2F2, N‐cadherin, and E‐cadherin expression levels in four groups of subcutaneously generated tumours as detected by IHC using serial slices.

Also, merely inhibiting miR‐214‐5p promotes cell proliferation (Figure 4A,B), migration, invasion (Figure 4C), tumour formation (Figure 5A,B), and tumour metastasis (Figure 5C,D). After that, we knocked down miR‐214‐5p in MATN1‐AS1 downregulated cells, and cancer malignant phenotypes were partly revised (Figure 4A–C, Figure 5A–D). IHC results from mouse xenograft tumour sequential slices showed similar trends (Figure 5E).

### 3.5 MATN1‐AS1/E2F2 Axis Contributes to Sunitinib Resistance in ccRCC

**FIGURE 6:**
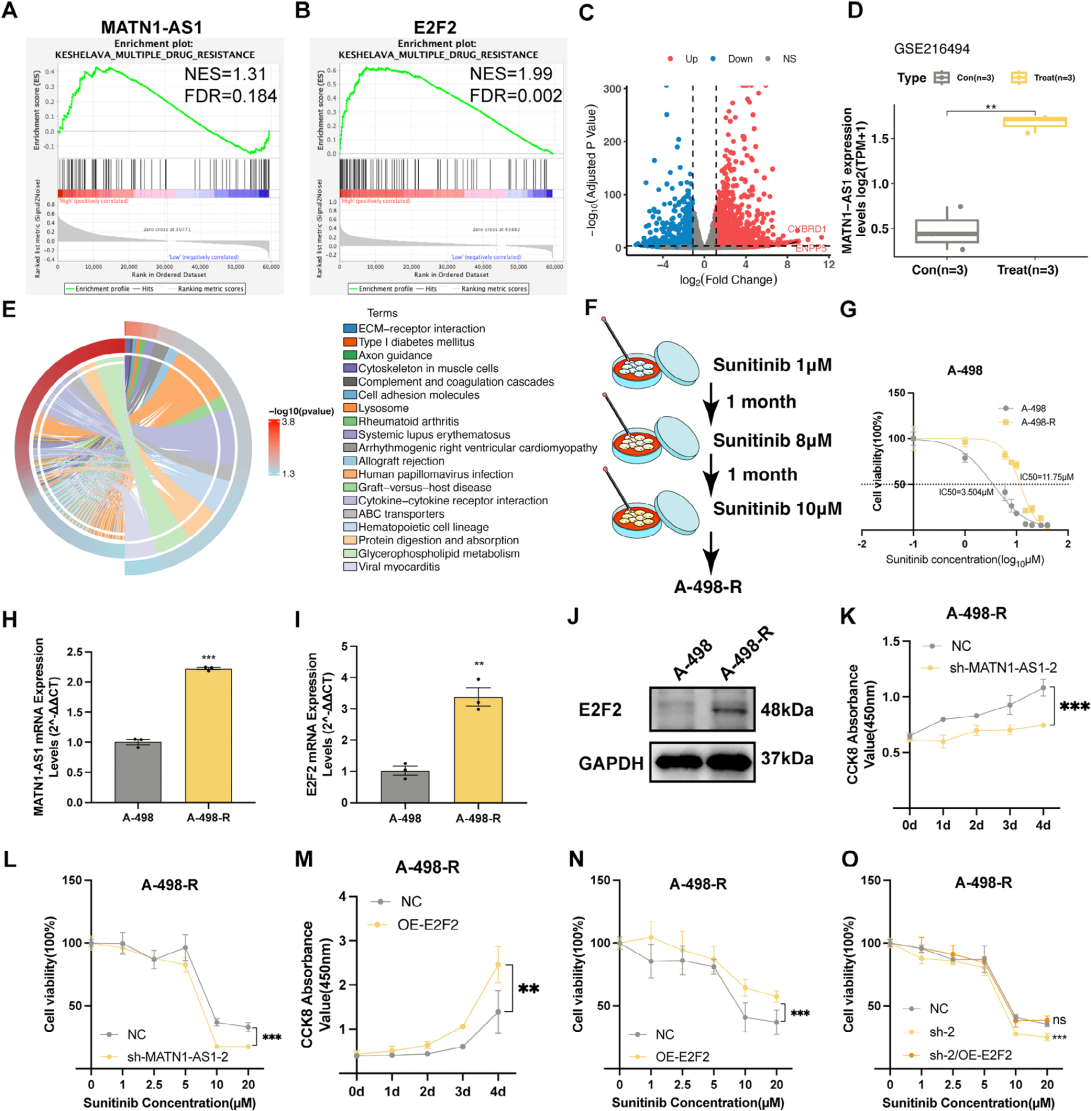
Knocking down MATN1‐AS1 reversed drug resistance in sunitinib resistance ccRCC. (A, B) GSEA of drug resistance signalling pathway. (C) DEGs between sunitinib resistance ccRCC cells and non‐treated cells of the GSE216494 dataset (|FDR| < 1, *p* < 0.05 was defined as non‐significance expressed genes filter criteria). (D) MATN1‐AS1 expression levels between sunitinib resistance ccRCC cells and non‐treated cells. (E) Gene ontology analysis of DEGs between sunitinib resistance ccRCC cells and non‐treated cells. (F) Graph diagram of sunitinib resistance A‐498 cell line (A‐498‐R) establishing process. (G) IC50 curves of sunitinib in A‐498 and A‐498‐R cell lines. (Data were presented as mean ± SEM). (H, I) MATN1‐AS1 and E2F2 RNA expression values between A‐498 and A‐498‐R cell lines (Mean ± SEM, Student's *t*‐test, **p < 0.01, ***p < 0.001). (J) E2F2 expression levels of A‐498 and A‐498‐R cells. (K) Knocking down MATN1‐AS1 inhibited cell viability of the A‐498‐R cell line cultured in 10 μM sunitinib (Mean ± SEM, Two‐way ANOVA, ***p < 0.001). (L) Drug‐response curves of sunitinib in MATN1‐AS1 knocked down A‐498‐R cells and non‐treated A‐498‐R cells (Mean ± SEM, Two‐way ANOVA test, ***p < 0.001). (M) Overexpression E2F2 reversed cell viability of the A‐498‐R cell line treated with 10 μM sunitinib (Mean ± SEM, Two‐way ANOVA, **p < 0.01). (N) Drug‐response curves of sunitinib in E2F2 overexpressed A‐498‐R cells and non‐treated A‐498‐R cells (Mean ± SEM, Two‐way ANOVA test, ***p < 0.001). (O) Drug‐response curves of sunitinib in indicated groups (Mean ± SEM, Two‐way ANOVA test, ns, no significance, ***p < 0.001).

In advanced renal cancer, tyrosine kinase inhibitor (TKI) therapy stands out to be the first‐line treatment. Previous studies have mentioned the unique role of EMT in promoting drug resistance [15]. Since the link between MATN1‐AS1/E2F2 and EMT, we are speculating on the underlying effect of the MATN1‐AS1/E2F2 axis in sunitinib (a classic TKI in RCC treatment) resistance. According to the GSEA analysis, the drug resistance signalling pathway was enriched in the MATN1‐AS1 and E2F2 high‐expression individuals (Figure 6A,B). In a previously conducted sequence on sunitinib resistance ccRCC cell line, we noticed an extreme upregulation of MATN1‐AS1 in DEGs (Figure 6C,D). Interestingly, the cell adhesion molecules pathway was altered between sunitinib resistance cells and non‐treated cells (Figure 6E). Then, a sunitinib resistance cell line, named A‐498‐R, was conducted by gradually adding up sunitinib concentration in the culture medium of the A‐498 cell line (Figure 6F). Our CCK8 results showed a significant difference in the A‐498‐R cell line on sunitinib resistance compared to the untreated cells (Figure 6G). MATN1‐AS1 was significantly up‐regulated in sunitinib‐resistance cells (Figure 6H), and so was the E2F2 expression level (Figure 6I,J). We checked whether targeting the MATN1‐AS1/E2F2 axis could reverse sunitinib resistance in ccRCC. Indeed, downregulating MATN1‐AS1 inhibited cell viability and the sunitinib resistance effect of A‐498‐R (Figure 6K,L), while overexpressing E2F2 showed the opposite result (Figure 6M,N). Also, increasing the E2F2 expression level revised the sunitinib resistance effect in MATN1‐AS1 downregulated A‐498‐R cells (Figure 6O).

In conclusion, MATN1‐AS1 could promote E2F2‐mediated EMT and sunitinib resistance effect by sponging miR‐214‐5p in ccRCC.

## 4 Discussion

Our research examined the potential oncogene role of MATN1‐AS1 in ccRCC. First, by utilising bioinformatics analysis and RT‐qPCR, we identified that MATN1‐AS1 was overexpressed in ccRCC samples. Additionally, increased expression of MATN1‐AS1 was linked to a deteriorating prognosis. And downregulating MATN1‐AS1 curbed ccRCC cell viability, tumour formation, and metastasis. We have also confirmed that MATN1‐AS1 could modulate E2F2 expression by sponging miR‐214‐5p, thereby promoting EMT. Furthermore, we also found the MATN1‐AS1/miR‐214‐5p/E2F2 axis role in sunitinib resistance occurrence.

Recently, lncRNAs have gained increasing attention in various studies due to their complex regulating mechanisms [25]. By competing binding with miR‐34/miR‐499, lncARSR could increase sunitinib resistance in renal cancer [9]. LncRNA SNHG12 could activate CDCA3 expression by stabilising SP1, thereby promoting tumour progression [26]. As for MATN1‐AS1, it could act as a sponge RNA that inhibits miRNA function, which resulted in the upregulation of downstream oncogene in glioma and osteosarcoma [13, 14]. For MATN1‐AS1 in ccRCC, the mechanism may be similar. According to our FISH assay results, MATN1‐AS1 was mainly located in the cytoplasm. A previous study demonstrated that MATN1‐AS1 could interact with E2F6 and inhibit RELA expression [12]. While in our study, the underlying regulator role of MATN1‐AS1 on E2F2, another same family member as E2F6, was found.

E2F2 is classified as one of the E2F transcription factors. Traditionally, E2Fs were recognised to be cell cycle managers [27]. By binding with cell cycle regulatory element cyclin D1, E2F8 promotes hepatocellular carcinoma cell proliferation [28]. In breast cancer, E2F8 was also evident to be related to the cell cycle signalling pathway [29]. However, E2F functions are far beyond being a cell cycle manager. Recently, other critical roles of E2Fs in malignancies have been uncovered. It regulates cell invasion by activating EMT in ovarian and cervical cancer [30, 31]. In addition, E2F8 could upregulate CDCA2 expression by cooperating with E2F2, thereby reducing cellular ROS accumulation [32]. While Shen et al. investigated E2F1 as a lipid synthesis manager in ccRCC [33]. In our study, knockdown MATN1‐AS1 significantly changed EMT biomarker expression and inhibited tumour metastasis ability. This effect was achieved through the regulation of E2F2 expression. In detail, MATN1‐AS1 sponges a micro‐RNA, miR‐214‐5p, thereby upregulating E2F2 levels.

For advanced renal cancer patients, the use of tyrosine kinase inhibitors (TKIs), such as sunitinib, is an important therapeutic strategy. However, due to the high occurrence rate of sunitinib resistance, the therapeutic efficacy is not considerable [4, 5, 34]. Therefore, identifying potential targets for reverse sunitinib resistance is of great importance. Recently, emerging evidence indicated the involvement of lncRNAs in drug resistance occurrence [35]. By regulating autophagy, lncRNA HOTAIR can induce sunitinib resistance [36]. In ccRCC, depleting lncRNA MALAT1 reversed sunitinib resistance [37]. However, no previous research has reported the association of MATN1‐AS1 with drug resistance. In our study, MATN1‐AS1 was overexpressed in sunitinib‐resistant ccRCC cells, and knockdown MATN1‐AS1 increased sunitinib sensitivity. While overexpressing E2F2 in cancer cells displayed an opposite function. Several studies also emphasised the pivot function of E2F2 in conducting drug resistance. In prostate cancer, E2F2 activated CIT expression through the Skp2‐p27 axis and conducted androgen deprivation therapy resistance [38]. Reimer et al. found that E2F2 was associated with platinum resistance [39]. Miyamoto et al. furtherly noticed the overexpression of E2F2 in paclitaxel resistance ovarian cancer cells, and inhibiting E2F2 restored paclitaxel sensitivity [40]. As for hepatocellular cancer, Wang et al. proposed that lncRNA AC026401.3, OCT1 dimer, could activate the transcription of E2F2 and induce sorafenib resistance [41]. Interestingly, sorafenib is another kind of TKI. However, low E2F2 expression leaded PARP inhibitor therapy resistance in breast cancer [42], this entirely different result indicated the heterogeneity between malignancies and drug responses. In conclusion, our result indicated the potential of targeting the MATN1‐AS1/E2F2 signalling pathway in reversing sunitinib resistance in ccRCC.

## 5 Conclusion

Collectively, our research has established that MATN1‐AS1 could regulate E2F2 expression, therefore promoting EMT and tumour metastasis in ccRCC. Furthermore, targeting MATN1‐AS1 reversed sunitinib resistance. Consequently, the findings show that MATN1‐AS1 could be a viable therapeutic target for ccRCC.

## Author Contributions

**Haibing Xiao:** conceptualization (equal), investigation (equal). **Mintian Fei:** investigation (equal). **Qili Xu:** investigation (equal). **Yu Gao:** investigation (equal). **Rui Feng:** formal analysis (equal). **Chaozhao Liang:** formal analysis (equal). **Baojun Wang:** data curation (equal), visualization (equal). **Haolin Li:** conceptualization (equal), data curation (equal), visualization (equal), writing – review and editing (equal).

## Ethics Statement

This study followed the ethical guidelines of the Declaration of Helsinki and was approved by the Ethics Committee of Human Research of The First Affiliated Hospital of Anhui Medical University (PJ2019‐14‐22). Animal experiments were performed according to the US National Institutes of Health Guide for the Care and Use of Laboratory Animals and was approved by the Institutional Animal Care and Use Committee of Anhui Medical University (LLSC20221265).

## Consent

Informed consent was obtained from all participants.

## Conflicts of Interest

The authors declare no conflicts of interest.

## Supporting information


Table S1.

Table S2.

## Data Availability

The original data presented in the study are openly available in TCGA at https://portal.gdc.cancer.gov, GSE66272, ICGC at https://dcc.icgc.org, GSE216494. Normalised high‐throughput sequencing data of 786‐O cells treated with lentivirus targeting MATN1‐AS1 could be obtained from supporting information.
